# Analysis of Microbial Functions in the Rhizosphere Using a Metabolic-Network Based Framework for Metagenomics Interpretation

**DOI:** 10.3389/fmicb.2017.01606

**Published:** 2017-08-23

**Authors:** Shany Ofaim, Maya Ofek-Lalzar, Noa Sela, Jiandong Jinag, Yechezkel Kashi, Dror Minz, Shiri Freilich

**Affiliations:** ^1^Newe Ya’ar Research Center, Agricultural Research Organization Ramat Yishay, Israel; ^2^Faculty of Biotechnology and Food Engineering, Technion-Israel Institute of Technology Haifa, Israel; ^3^Institute of Soil, Water and Environmental Sciences, Agricultural Research Organization Beit Dagan, Israel; ^4^Department of Plant Pathology and Weed Research, Agricultural Research Organization, The Volcani Center Beit Dagan, Israel; ^5^Department of Microbiology, College of Life Sciences, Nanjing Agricultural University Nanjing, China

**Keywords:** metabolic networks, microbial community, rhizosphere, microbial ecology, computational analysis

## Abstract

Advances in metagenomics enable high resolution description of complex bacterial communities in their natural environments. Consequently, conceptual approaches for community level functional analysis are in high need. Here, we introduce a framework for a metagenomics-based analysis of community functions. Environment-specific gene catalogs, derived from metagenomes, are processed into metabolic-network representation. By applying established ecological conventions, network-edges (metabolic functions) are assigned with taxonomic annotations according to the dominance level of specific groups. Once a function-taxonomy link is established, prediction of the impact of dominant taxa on the overall community performances is assessed by simulating removal or addition of edges (taxa associated functions). This approach is demonstrated on metagenomic data describing the microbial communities from the root environment of two crop plants – wheat and cucumber. Predictions for environment-dependent effects revealed differences between treatments (root vs. soil), corresponding to documented observations. Metabolism of specific plant exudates (e.g., organic acids, flavonoids) was linked with distinct taxonomic groups in simulated root, but not soil, environments. These dependencies point to the impact of these metabolite families as determinants of community structure. Simulations of the activity of pairwise combinations of taxonomic groups (order level) predicted the possible production of complementary metabolites. Complementation profiles allow formulating a possible metabolic role for observed co-occurrence patterns. For example, production of tryptophan-associated metabolites through complementary interactions is unique to the tryptophan-deficient cucumber root environment. Our approach enables formulation of testable predictions for species contribution to community activity and exploration of the functional outcome of structural shifts in complex bacterial communities. Understanding community-level metabolism is an essential step toward the manipulation and optimization of microbial function. Here, we introduce an analysis framework addressing three key challenges of such data: producing quantified links between taxonomy and function; contextualizing discrete functions into communal networks; and simulating environmental impact on community performances. New technologies will soon provide a high-coverage description of biotic and a-biotic aspects of complex microbial communities such as these found in gut and soil. This framework was designed to allow the integration of high-throughput metabolomic and metagenomic data toward tackling the intricate associations between community structure, community function, and metabolic inputs.

## Introduction

The biology of individual organisms is linked to their community and ecosystems *via* metabolic activity. Organisms take up energy and resources from the environment, convert them into other forms, and excrete altered forms back into the environment (Brown et al., 2004; Perez-Garcia et al., 2016; Zomorrodi and Segre, 2016). Metabolic activity is a key determinant of interaction patterns between micro-organisms (Klitgord and Segre, 2011; Widder et al., 2016). Microbial species not only compete for the available resources, but in many cases work together toward the degradation of complex polymers into simpler compounds (Schink, 2002; Fuhrman, 2009; Marx, 2009; Koropatkin et al., 2012; Grosskopf and Soyer, 2014). Degradation chains shape the structure of the community as a primary degrader mediates the accessibility of energy sources to other members of the community. Secondary degraders rely on the presence of the primary mediators, and the final excretion products are determined according to the identity of the downstream chain members. The perception of ecosystems as a trinity of environment (specific resources) – community (possible conversion repertoire) – and function (excretion of altered forms), provides a conceptual framework for the study of microbial activity in ecological habitats. Shifts in community structure are hence assumed to reflect changes in either one or both adjacent edges in the community-environment-function trinity.

High-resolution mapping of shifts in bacterial community structure has become widely accessible with the development of massive, low-cost, sequencing techniques. Together with biodiversity detection in environmental samples, metagenomics projects allow the construction of community-level gene catalogs (Zengler and Palsson, 2012; Franzosa et al., 2015; Guo et al., 2015; Widder et al., 2016). A considerable effort has been invested in the development of computational approaches for a functional-oriented interpretation of such data and specifically in deciphering the variations in metabolic activity between treatments (Stolyar et al., 2007; Freilich et al., 2011; O’Dwyer et al., 2012; Segata et al., 2013; Roling and van Bodegom, 2014; Zomorrodi et al., 2014; Bowman and Ducklow, 2015; Guo et al., 2015; Hanemaaijer et al., 2015; Roume et al., 2015; Zelezniak et al., 2015; Granger et al., 2016). Metagenomics driven gene catalogs are typically two dimensional, i.e., genes can be classified according to both functional annotation and taxonomic affiliation (Greenblum et al., 2012). Functional annotations allow the construction of community level metabolic networks, similarly to the construction of species-specific networks, based on the content of enzyme coding genes in their respective genomes (Abubucker et al., 2012; Levy and Borenstein, 2013; Roume et al., 2015; Tobalina et al., 2015). Subsequently, predictions for network-specific sets of source-metabolites can be inferred through computational approaches, providing an approximation of the relevant metabolic content of an environment (Borenstein et al., 2008; Handorf et al., 2008). Computational simulations can then address the influence of environmental inputs (nutritional resources) on network dynamics. At the species (genome) level, metabolic-activity simulation allows predicting the effects of environmental and genetic perturbations through iterative modifications of the available metabolic inputs and/or network structure, respectively (Freilich et al., 2009, 2010). At the community level, a similar approach can be applied for delineating functional division between community members. By overlaying the taxonomic dimension over network edges (functional annotation), metabolic capacities contributed exclusively by specific taxa can be grouped. The communal network functional performances can then be tested by simulating the iterative removal or addition of corresponding network edges specifically associated with key taxonomic groups. Such iterations can, first, describe the metabolic hierarchy where different taxonomic groups are expected to vary in their contribution for converting complex nutrients into widely accessible ones; second, reveal variations between treatments in network performances.

The main goal of this study is to use metabolic-network approach to explore the environment-function-structure associations in the complex microbial communities of the rhizosphere microbiome (rhizobiome). The rhizosphere is the soil known as the area that is directly under the influence of living roots. The rhizobiome is known to be strongly influenced by plant roots activity. These act as selective nutritional sources for phytochemicals that stimulate and support enrichment of specific groups of soil microorganisms (Larkin et al., 1993; Smith et al., 1997, 1999; Berg et al., 2002; Mazzola, 2004; Cook, 2006; Ikeda et al., 2006; Micallef et al., 2009; Ofek-Lalzar et al., 2014; Ofek et al., 2014; Haldar and Sengupta, 2015). Advances in sequencing technologies promoted the extensive characterization of community structures in rhizosphere compared with the more distant soil, not under the direct effect of the root (Mirete and González-Pastor, 2010; Turner et al., 2013; Bouffaud et al., 2014; Lakshmanan et al., 2014; Ofek et al., 2014; Roume et al., 2015). A published gene catalog, constructed from genomic DNA that was extracted from the root and respective soil samples of cucumber and wheat, was used for characterizing a core set of functional genes associated with root colonization (Ofek-Lalzar et al., 2014). Here, we hypothesized that analyzing this gene catalog using a metabolic network based framework will further allow associating specific functions with taxonomic groups and external metabolic signals (such as those induced by root plants). Starting from this gene catalog, we constructed four environment-specific metabolic networks (cucumber root and soil; wheat root and soil) and predicted specific externally consumed metabolites associated with each environment, as well as network functions dominated by specific taxonomic groups (order level). The impact of each taxonomic group was assessed through the dynamic removal of the enzymatic functions of specific groups, one by one and all at once. Similarly, complementation potential of bacterial combinations – that is, the ability of taxonomic groups to co-produce metabolites that are not synthesized by any of the individual entities, was explored in the four different niches.

## Materials and Methods

### Metagenomic Data, Samples, Sequencing, and Annotations

The metagenomics-derived gene catalogs used for the current analysis was previously reported in Ofek-Lalzar et al. (2014) and is publically available (BioProject accession number PRJNA208116). In brief, the DNA data were extracted from two agricultural crops: wheat and cucumber. For each crop plant, samples were taken from rhizosphere – the area under the direct influence of the root, and the more distant soil not under direct effect, termed here root and soil samples, respectively. The replicated experiment included 10 samples in total: root samples were in triplicates (a total of six root samples) and soil samples in duplicates (a total of four samples). Reproducibility between replicas was tested and reported, clearly demonstrating higher variance between root and soil treatments. The data were sequenced, annotated and mapped to taxonomic bins and KEGG ortholog groups. Taxonomic assignments were done using the lowest common ancestor algorithm, MEGAN (version 4.0) (Huson et al., 2007). The gene catalog represents approximately 71% of cucumber root reads, 50% of wheat root reads and 34% of soil reads. DNA reads were mapped to different taxonomic ranks. Approximately 72, 63, and 47% of the reads in soil cucumber and wheat samples were mapped to bacteria, while 22, 16, and 24% were ‘not assigned,’ respectively. Generally, out of the total number of reads mapped to bacteria, over 97% of the reads mapped to the bacterial level were assigned to order level. Overall, this gene catalog provides a description of genes detected in each of the four treatments (cucumber root and soil; wheat root and soil), their relative abundance, functional annotation (e.g., KEGG assignment), and taxonomic origin. This gene catalog was used as a starting point for network construction and subsequent analyses.

### Network Construction

KEGG ortholog groups associated with enzymatic functions were detected across the four environments. Differential abundance (root vs. soil) of enzyme-associated reads was determined independently for wheat and cucumber using the EdgeR R package (Robinson et al., 2010) under the set of conditions previously described by Ofek-Lalzar et al. (2014). Differential abundance between environments was considered significant if the difference was greater than two fold and the FDR-adjusted *p*-value was < 0.01, requiring consistency between replicas. Overall, the differential abundance analysis produced four sets of differentially abundant enzyme sets describing: cucumber soil, cucumber root, wheat soil and wheat root (**Supplementary Tables S1, S2** and **Figure S1**). In each environment, the set of differentially abundant enzymes was used for construction of an environment-specific network, following the procedure outlined in Kreimer et al. (2012). Similarly, a meta-network was constructed, containing all enzymatic functions annotated across the metagenomic data.

### Prediction of Environment-Specific Metabolites (Source-Metabolites)

Using the NetSeed algorithm (Carr and Borenstein, 2012), through its implantation in NetCmpt (Kreimer et al., 2012), an approximation of the relevant metabolic environment was retrieved for each of the five networks (meta-network and four environment-specific networks). Based on network topology, the algorithm provided a list of metabolites that were predicted to be externally consumed from the environment, termed here ‘source metabolites.’ Since the four environment-specific networks were constructed from differentially abundant enzymes only, they were highly fragmented, leading to prediction of artificial source-metabolites (**Supplementary Figure S2**). Source-metabolites, identified for each environment specific network, were hence compared to the source-metabolite set identified for the meta-network. Only source-metabolites present in both sets were further considered. Following this filtration, we complied four environment-specific sets of source metabolites providing an approximation of the metabolic content in the corresponding environments (root and soil of wheat and cucumber, a total of four environments, **Supplementary Tables S3, S4** and **Figure S1**).

### Network Expansion Algorithm and Its Application for Describing Environmental Activity

To predict metabolic activities in each environment we made use of the Expansion algorithm (Ebenhoh et al., 2004). Briefly, the algorithm allows the predicting of an active metabolic network (expanded) given a pre-defined set of substrates and reactions. The algorithm starts with a set of source-metabolites acting as substrates; it scans the reaction bank for feasible reactions for which all the possible substrates exits; all feasible reactions are added to the network, their products being the substrates for the next set of reactions. The network stops expanding when no feasible reactions are found. Thus, the full expansion of the network reflects both the reaction repertoire and the primary set of compounds (source-metabolites). Here, simulations of environmental activity were carried by expanding the meta-network (the full set of reaction detected across all samples) four times – each time using the environmental specific source metabolite set. We made use of the full set of reactions for all simulations, since despite differences in abundance, almost all enzymes were detected in all samples (Ofek-Lalzar et al., 2014). The environment-specific expanded networks are provided at **Supplementary Table S5**.

### Taxonomic Mapping and Dominance Establishment

All sequenced reads, collected in the metagenomic dataset, were linked to taxonomic groups, in the order level, using mapping from previous analysis (Ofek-Lalzar et al., 2014). Each read was assigned a Gene Id which was used as key parameter. Enzymes were then linked to the taxonomy mapping through gene IDs. The Simpson index (Heip et al., 1998), typically used to determine species dominance in ecological surveys, was newly applied here to determine the dominance of specific taxonomic groups in regards to a function. To this end, instead of looking at the frequencies of species in a sample (as in ecological surveys), for each enzyme (equivalent to a sample), we looked at the distribution the taxonomic affiliations of its associated reads. Accordingly – a low score denoted a function carried by many taxonomic groups; high score denoted a function carried out by single or few groups. To this end, Simpson Indices were calculated for each enzyme in the dataset for each of the original 10 samples (**Supplementary Table S6**). Replicates show similarity in the dominance/diversity indices (**Supplementary Figure S3**). Then, an environmental Simpson index value was determined for each enzyme by calculating mean values across corresponding samples. Finally, within each environment, we described a function to be dominated by a taxonomic group if: (i) the environmental Simpson index value was greater than 0.4 – mean dominance value across all enzymes (**Supplementary Table S6**) typically also associated with low diversity (**Supplementary Figure S3**); (ii) the same taxonomic group is dominate in all replicate samples. Hence, associations between taxonomy are representative of a treatment (environment) and consistent between replicas. Most (556) of the dominant enzymes were associated with a single taxonomic group (that is, dominance by a single taxonomic groups in different environments; **Supplementary Table S7**). For each environment, we ranked taxonomic groups according to the number of enzymes they dominate. Top five groups were termed ’key’ taxonomic groups.

### Dynamic Removal and Functional Analysis

For each environment, network expansions were carried six times using the corresponding set of source metabolites. In the first of the six iterations, the reaction set included the full set of metabolic functions (as described above). In each of the subsequent five iterations – all edges (metabolic functions) specifically dominated by one of the key taxonomic groups were removed from the original enzyme set. The impact of the removal of each key taxonomic group was estimated according to differences in the metabolite content (metabolite number) between the network expanded from the truncated enzyme set, and the original meta-network (first iteration, expanded from the full enzymatic set). The removed metabolite vectors, created for each iteration, were mapped to KEGG pathways. A removal effect score was calculated for each pathway as the fraction of metabolites left after the removal out of the original number of metabolites per pathway (counted in the first iteration, considering the un-truncated network).

### Synergistic Metabolic Complementation

In order to discover whether there is a synergistic metabolic complementation between combinations of key taxonomic groups, we applied a reverse approach to the removal procedure described above. Starting from a core set of enzymes representing common functions (i.e., functions that are not dominated by a key taxonomic group) we added combinations of specific and unique taxa-dominated enzyme sets. The possible combinations are described in **Supplementary Table S8**. An enzyme set was automatically created for each possible combination in each environment, according to the Simpson dominance scores. Thus leading to, for each combination tested, a number of enzyme sets as the number of combination members in addition to the enzyme set describing the amalgamate. Each enzyme set was used to expand a network as previously described. Each combination-type was tested in all four environments. All the networks were scanned for complementary metabolites. To identify complementary metabolites, we compared the metabolite content of multi-members networks to these of its corresponding single-member networks. That is, for a combination of a pair of taxonomic groups A and B, we expanded three networks per environment, giving a total of three networks i.e., (1) a core network + enzymes dominated by group A; (2) a core network + enzymes dominated by group B and (3) a core network + enzymes dominated by groups A and B (**Supplementary Figure S4**). Metabolites that were produced in the joint network (network 3), but not in the individual networks (networks 1 and 2), were termed complementary metabolites. For each such combination, the process was carried in each of the four environments. All complementary metabolites were than mapped to KEGG pathways (**Supplementary Table S9**).

### Pathway Mapping and Enrichment Analysis

Enzymes and metabolites were cataloged and mapped to pathways according to the KEGG database (Kanehisa et al., 2014). Testing for significantly enriched pathways (metabolites/enzymes) was done using the results of the hypergeometric enrichment test as in Yadav et al. (2016). In addition, using the R chisq.test function (R version 3.2.2), the X^2^ goodness of fit test was used to evaluate the compliance of subsets with the relative distribution of samples. A pathway was considered significantly enriched if it passed both the hypergeometric distribution (*p* < 0.05) and the X^2^ goodness of fit (*p* < 0.05) tests.

### Visualizations

Venn diagrams were made using Venny (Oliveros, 2007) then adapted using Microsoft VISIO 2013. PCA analysis was performed using R prcomp function (R version 3.2.2). Heatmap and PCA plots were made using the ggplot2 R package (version 1.0.1) (Ito and Murphy, 2013). Pathways were plotted into a heatmap using the pheatmap R package (version 1.0.8) (Kolde, 2015). All network visualizations were made using Cytoscape (version 3.3.0) (Shannon et al., 2003).

## Results

Here, we present a framework for the analysis of functionally and taxonomically annotated metagenomic data. In brief, the main steps of the framework are the following (1) the construction of a general metagenomic network (termed meta-network), used as reference network, and treatment specific networks; (2) *in silico* predictions of source-metabolite sets to be used to describe the metabolic content of the corresponding environments; (3) establishing a link between functions, taxonomy groups and DNA reads using an ecological element, the Simpson index, per environment; (4) dynamic removal of sets of unique enzymes specific to each key taxonomic groups; and (5) assessment of taxonomic group complementation (or synergism).

### Comparative Analyses of Environmental Metabolic Functions

**FIGURE 1:**
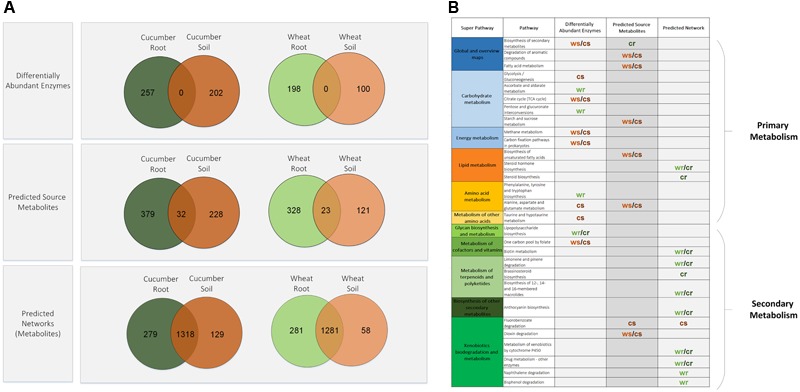
Distribution patterns of differentially abundant enzymes, source-metabolites and network metabolites from the four experimental environments. **(A)** Venn diagrams of root versus soil entities. **(B)** Pathway significantly enriched with root versus soil entities. cr, cucumber root; cs, cucumber soil; wr, wheat root; ws, wheat soil.

Gene catalogs used here were constructed based on published DNA metagenomic data that were collected from four environments – roots of wheat and cucumber and the corresponding soils (Ofek-Lalzar et al., 2014). A total of 3436 KEGG orthologs, identified across all data, were mapped to 1574 unique enzymes (denoted by a four digits EC numbers) which represent the overall cross-environment compilation. We first identified differentially abundant enzymes in soil vs. root environments, considering each crop independently. Both plant treatments showed an overall similarity in their root vs. soil divergence pattern with most of the differentially abundant enzymes shared by both crops (**Figure 1A**). The differentially abundant enzyme sets were mapped to 100 KEGG pathways (**Supplementary Table S10**), with only nine showing significant enrichment in a specific environment (**Figure 1B**). Enrichment pattern of functional categories corresponds with previous reports: while some of the enriched root associated pathways were found to be involved in lipopolysaccharide metabolism, in agreement with Owen et al. (2007) and Ofek-Lalzar et al. (2014), most of enriched soil associated pathways were mostly of primary metabolism such as the TCA cycle and carbon metabolism. Next step was going beyond the list of discrete genes and integrating data into networks, according to stages framework outlined above. The sets of environment specific enzymes were used for the construction of four corresponding environment-specific networks. Subsequently, computationally based approximations of each metabolic environment were calculated based on the network topology. Similarly to the differentially abundant enzyme groups, most of the predicted source-metabolites in the root and soil environments are shared by both crops (**Figure 1A**). The source-metabolites were mapped to 90 KEGG pathways (**Supplementary Table S11**). When comparing the pathway distribution of source-metabolites across the different environments, seven pathways showing significant environment-specific associations were detected (**Figure 1B**). Most of the significant pathways were identified for the soil environments, with the exception of pathways from the biosynthesis of secondary metabolites category, that were significantly enriched in the cucumber root environment (**Figure 1B**), possibly reflecting the effect of root exudates.

### Comparative Analyses of Environment-Specific Community Metabolic-Networks

The description of the predicted source-metabolites, together with the metabolic potential (the enzymes), allowed simulating metabolic activity in each of the four environments. As can be expected in natural robust systems (Freilich et al., 2010), the large majority of basic metabolism was carried despite environmental variations (**Figure 1A** bottom, and **Supplementary Table S12**). To delineate environmental induced metabolic activity, network metabolites were mapped to 122 KEGG pathways. Pathway mapping of the expanded networks showed differences in a wide range of secondary metabolism functions (**Figure 1B**). This stood in contrast to enriched pathways found in the initial enzymes and source-metabolites sets, where differences were found mainly in primary metabolic functions. Most of the divergent pathways found for the environment-specific networks were associated with root environments (**Figure 1B**). Some of these root-enriched pathways, belong to secondary metabolism categories, including metabolism of terpenoids, polyketides, and anthocyanins. These, are common plant metabolites that are less likely to be abundant with increasing distance from the root (Owen et al., 2007; Megharaj et al., 2011; Jeon and Madsen, 2013; Jha et al., 2015). These root unique network functions support the ecological relevance of the expanded environment-specific networks and their relevance for delineating robust versus unique metabolic capacities.

### Taxa-Dominated Functions and Their Contribution to Communal Performances

**FIGURE 2:**
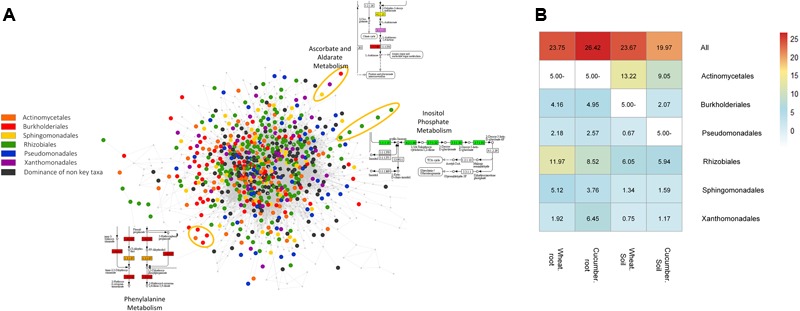
Network representation and performances of enzymes dominated by key taxonomic groups. **(A)** Network representation of dominated versus non-dominated enzymatic functions. Nodes in the network represent enzymes linked according to subsequent reactions. Core enzymes, that are, enzymes that are not dominated by any taxonomic group and are common to multiple groups are marked in gray; dominated enzymes are colored. Examples for network areas of specific pathways are circled in different colors. **(B)** Impact of the removal of key taxonomic groups, one by one and all at once on network metabolite content (number). Colored values denote the percentage of missing metabolites in the network following removal out of the total number of metabolites in the reference network without removal. The ’All’ row denotes the removal of all key groups across environments. No available data is denoted in empty white squares indicating that the corresponding group was not one of the top key dominant groups in the corresponding sample.

Considering the triangular relationship between an organism, a function and an environment, we set out to project taxonomy information over the environment-specific networks. In each environment, enzymes were scored according to the taxonomic diversity of their associated reads. A total of 667 (out of 1574) enzymes were paired with dominant taxa (order-level) in at least a single environment. Most differences in the classification profiles of these sets were associated with relative representation in secondary metabolism categories (**Supplementary Figure S5**). In each environment, the five taxonomic groups with the highest number of dominated enzymes were defined as key taxonomic groups (**Supplementary Table S13** and **Figure S6**). Overall, these key taxonomic groups were similar across the four environments and included *Actinomycetales* (in soil environments only), *Burkholderiales, Pseudomonadales, Rhizobiales, Sphingomonadales*, and *Xanthomonadales*. Conserved vs. dominated functions in the communal metabolic network of the cucumber root are illustrated in **Figure 2A**. To directly explore the contribution of taxa-dominated functions to community performances, we simulated network activity while eliminating such enzymes, all at once and group by group (Methods, **Supplementary Table S14**). This iterative removal-expansion process directly explored the impact of the key taxonomic groups on metabolic processes carried in their specific environment. In general, the effect of function removal was found to depend both on its hierarchical positioning in a pathway (for example – an enzyme converting a source-metabolite into a compound accessible for multiple groups in the community will have a high impact), and on the robustness of the pathway (the prospects of finding alternative routes for the production of the corresponding metabolites). The impact is only relevant to network studies, representing a snapshot of community structure at a given time point. Despite the robustness of the expanded metabolic networks, the removal of all key taxonomic group specific functions led to up to approximately 26% reduction in network size (Methods, **Figure 2B**). The highest removal impact was observed in the cucumber root environment. In general, the *Rhizobiales* and the *Actinomycetales* groups have had the highest removal impact in root and the soil environments, respectively (**Figure 2B**). These taxonomic groups are generally known to be abundant and highly dominant in soil and root environments (Mendes et al., 2013; Tian and Gao, 2014).

### Delineation of Environment-Taxa-Function Associations

**FIGURE 3:**
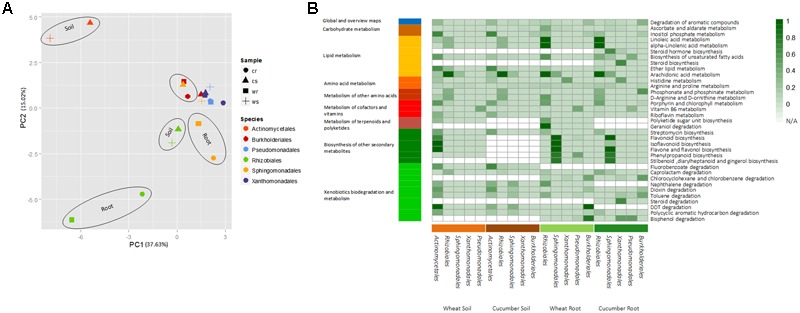
The removal effect of enzymes dominated by key taxonomic groups on the metabolic potential of the community. **(A)** PCA analysis plot of the removal effect of taxonomic groups in different environments on the overall metabolic capacity of the corresponding communities. Vector profiles describe the absence/presence of metabolites in the absence/presence of the corresponding group. **(B)** Mapping of missing metabolites into specific pathways. Square color strength (white to dark green) denotes the degree of contribution of the taxonomic group to the denoted function, i.e., the fraction of missing metabolites following removal of the corresponding taxonomic groups out of all metabolites in the pathways without removal. White squares denote non-available data per function per sample indicating that the corresponding group was not one of the top key dominant groups in the corresponding sample. Only pathways with an effect score greater than 0.4 in at least a single experiment (removal iteration) are shown. The full removal description is available at **Supplementary Tables S15–S18**.

The metabolic processes affected by the removal of key taxonomic group (order level) were delineated by mapping the omitted metabolites into KEGG pathways (Methods, **Supplementary Tables S15–S18**). As expected in ecological systems, the removal effect was observed to be both group specific, environment dependent and differed between root and soil (**Figure 3A**). Pathway mapping has shown that the large majority of pathways affected were belongs to secondary-metabolism categories (**Figure 3B**). In the soil, the removal of the order *Actinomycetales* invoked the highest predicted impact. One example for an affected function is streptomycin biosynthesis (**Figure 3B**), an antibiotic produced by bacteria from the order *Actinomycetales* (Nett et al., 2009; Zhou et al., 2012; Mitter et al., 2013; Panov et al., 2013). *Actinomycetales’* significant impact was also simulated in various pathways associated with degradation of fluorobenzoate and compounds from the polychlorinated biphenyls (PCBs) pollutant family. This may stem from one of *Actinomycetales* high dominance enzyme, benzoate 1,2-dioxygenase (EC 1.14.12.10), which catalyzes a variety of ’first-step’ reactions toward the degradation of an array of benzoate analogs (Solyanikova et al., 2015). In the root environment, pathways that were affected by the removal included lipids, terepnoids, and plant induced secondary metabolites categories (**Figure 3B**). The taxonomic group with the highest impact in the root, in accordance with its key role in the rhizosphere (Berg and Smalla, 2009), was the *Rhizobiales* (**Figure 3B**). Out of 14 reactions involved in arachidonic acid metabolism, only a single reaction (aryl-4-monooxygenase) was simulated to be highly dominant by *Rhizobiales* sequences. However, its upstream location in the pathway suggests that *Rhizobiales* may be crucial for its metabolism in the surveyed environment. In addition, the simulated removal of *Rhizobiales* in the root (but not in the soil) affected the metabolism of linoleic acid and geraniol associated pathways. Both compounds are plant exudates that are used as carbon sources in the rhizosphere (Folman et al., 2001; Owen et al., 2007). Similarly, the simulated removal of *Sphingomonadales* in the root (but not in soil) affected mostly phenylpropanoid and flavonoid-related pathways (**Figure 3B**). These root-specific effects correspond with the role of plant exudates such as flavonoids, organic acids, and carbohydrates as determinants of the microorganism community structure in the rhizosphere (Narasimhan et al., 2003; Schulz and Dickschat, 2007; Ofek-Lalzar et al., 2014). Bisphenol degradation in the root, was affected by the removal of *Actinomycetales, Pseudomonadales* and *Burkholderiale*, but not by *Rhizobiales*, in correspondence with recent reports (Matsumura et al., 2015). Other pathway categories uniquely affected included these involved in the metabolism of potential regulators of plant–microbe interactions. For example, vitamin B6 was uniquely affected by the removal of the *Pseudomonadales* taxonomic group (**Figure 3B**), in accordance with their role in the production of B- group vitamins in the rhizosphere (Marek-Kozaczuk and Skorupska, 2001).

### Relating Co-occurrence Patterns to Metabolic Exchange Interactions

**FIGURE 4:**
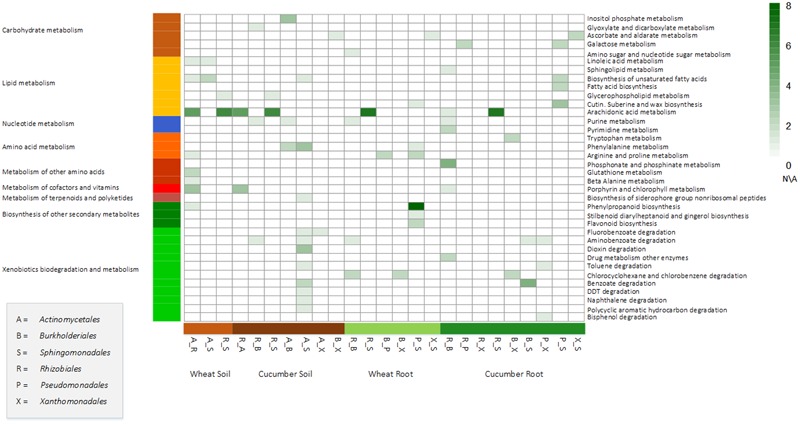
Mapping of complementary metabolites into specific pathways. Complementary metabolites are these created in the network when adding enzymes dominated by both members of a pairwise combinations to a core network of non-dominated enzymes and are not formed when only adding the enzymes dominated by one of the pair members. Square strength color (white to dark green) denotes the number complementary metabolites per combination in a specific environment. White squares denote non-available data per function per sample indicating that the corresponding group was not one of the top key dominant groups in the corresponding sample.

Bacterial communities, or combinations of taxonomic groups, are suggested to perform tasks that no species could perform on their individually (Freilich et al., 2011; Zomorrodi and Segre, 2016). Here, a simulative system was applied for surveying such potential synergistic interactions between the key taxonomic groups. A synergistic interaction was estimated according to complementary metabolites, defined as metabolites that are produced only in the presence of a combination of species, and not by individual members of the combination. These, environment-specific, combination-specific complementary metabolites were mapped to pathways (**Figure 4**, methods). Many of these predicted processes aligned well with ecological theories, hence providing a functional rational to observed co-occurrence patterns.

The highest number of complementary metabolites was simulated for a combination of *Pseuodomonadales* and *Sphingomonadales* in the wheat root environment (20 metabolites, **Supplementary Table S19**). Bacteria from these two taxonomic groups were demonstrated as having a co-dependent distribution pattern across environments (Pascual-Garcia et al., 2014). Most of the complementary metabolites predicted for the *Pseuodomonadales*–*Sphingomonadales* combination were mapped to the phenylpropanoid biosynthesis pathway (**Figure 4**). This corresponds with the demonstrated activity of *Pseudomonadales* in the rhizosphere that *via* the phenylpropanoid pathway assist the plants’ response to biotic stresses by contributing to a consortium that elicits the accumulation of phenolic compounds (Jain et al., 2012; Singh et al., 2013). Complementary metabolites predicted to be produced by a *Actinomycetales*–*Burkholderiales* combination included those in the pathway of inositol-phosphate metabolism (**Figure 4**). This corresponds with the suggested role of rhizobacteria in increasing phosphorus availability in the rhizosphere, hence contributing to plant growth (Unno et al., 2005; Singh and Satyanarayana, 2011; Wang et al., 2013). *Burkholderiales* and *Xanthomonadales* showed a simulated synergistic complementary effect related to tryptophan metabolism in the cucumber root environment. Tryptophan, secreted by the root, is converted by rhizobacteria to auxin, an hormone promoting plant growth (Kamilova et al., 2006). In the cucumber rhizosphere, amounts of secreted tryptophan were reported to be low in comparison to other crops (Kamilova et al., 2006). Hence, this cucumber-root specific synergistic complementary predicted effect may indicate a unique adaptation to a low tryptophan environment. Finally, complementation between *Actinomycetales* and *Sphingomonadales* was simulated in several pathways associated with PCB degradation and especially in dioxin degradation. As mentioned above, *Actinomycetales* dominate the ‘first step’ catalyzing enzyme. Bacteria from *Sphingomonadales* order dominate downstream enzymes. Thus the presence of both taxonomic groups, in turn, may lead to the use of PCBs as a carbon source.

## Discussion

“Omics” approaches are moving toward describing the full picture of host–microbe interactions requiring integration and systems-level modeling (Nayfach and Pollard, 2016). Here, we suggest a framework for the functional interpretation of metagenomic data providing predictions for the contribution of key taxonomic groups to the overall community performances. Identification of such group-specific functions is typically not trivial and is hampered by the complex nature of microbial communities. Our approach addresses three key challenges. First, almost all functions are associated, to different degrees, with multiple taxonomic groups. Hence, the definition of unique *vs.* core enzymes requires quantitative estimates for the phylogenetic representation of reads assigned. This need for a measure of the degree of taxonomic dominance over function can be viewed as an extension of the long-discussed concept of taxonomic dominance over ecological environments. The Simpson index is the conventional measure used by ecologists to describe species dominance in a habitat (Heip et al., 1998; Zhang et al., 2014). The innovative application of this well-established index in the current study produced quantified links between functions and taxonomic groups, tackling an unsolved challenge in functional analysis. Once such link is established, a second challenge is predicting the impact of taxa-dominated functions on overall community performance. It is well-established that environments populated by highly complex bacterial communities show high functional robustness (Monard et al., 2011; Stenuit and Agathos, 2015; Bordron et al., 2016). The contextualizing of discrete enzymes into functional networks, as done here, allows directly assessing robust functions vs. these relying on specific groups/group combinations. Third, taxonomic structure and functional variations are often induced by environmental inputs. Our framework allows an approximation of environmental effect through simulating activity in different natural-like environments.

Here, we demonstrate the application of the framework for the analysis of a metagenomics derived gene catalog from the complex microbial communities of plant roots (Ofek-Lalzar et al., 2014). The rhizobiome is a central determinant of crop health and yield, hence understanding how to manipulate rhziobiome communities toward desired function is a major agricultural concern (Mazzola and Freilich, 2017). Rhizobiome communities are strongly influenced by root activity where plant secretion is a key determinant of their structure (De-la-Pena and Loyola-Vargas, 2014; Jha et al., 2015). Our framework was applied for tackling the intricate associations between community structure, community function, and metabolic inputs in this important ecosystem. The metabolic context created by these associations extends previous findings of functional capabilities in root systems and allows testing the significance of individual taxonomic groups within their community. We simulated environment specific communal performances, associated functions with specific taxonomic groups, and identified potential co-exchange patterns leading to the production of complementary metabolites. The simulations and the resulting predictions are environment-specific, based on computational approximation of the key available nutrients in different treatments. The simulated observations are in accordance with common ecological and network concepts. First, the communal networks are highly robust where the large majority of basic metabolism functions are conserved between environment and do not rely on specific groups. Yet, despite this inherent robustness, the analysis succeeded in pointing at several taxonomic-associated functions. Many of these functions are unique to the root-like environment (vs. soil) and are in agreement with reported observations. Examples for such predictions made include utilization of plant exudates as linoleic-acid, flavonoids, and geraniol by *Rhizobiales, Sphingomonadales*, and *Burkholderiales*. Finally, the predictions for the profiles of complementary metabolites, formed between specific taxonomic combinations in specific environments, may suggest a possible functional significance for observed co-occurrence patterns. For example, *Burkholderiales* and *Xanthomonadales* activity can possibly compensate for the low levels of tryptophan secreted in by the cucumber’s root.

Overall, the presented approach was successful in predicting root-specific effects that link the utilization of specific environmental nutrients (here, plant exudates) with specific taxonomic groups, pointing at the impact of each such compound as a determinant of the microbial community structure. Caveats of the current analysis, reflecting both data-driven and conceptual limitations should be acknowledged. Most notably, data-driven limitations include the partial coverage of metagenomic sequence data. The dataset used here, as in most data collected from complex environments (such as the root and soil), does not provide a full coverage description of the corresponding communities. Future projects are expected to provide a rapidly increasing coverage; such coverage will allow the detection of the less abundant functions and assembly guided taxonomic classification of sequence reads. In parallel to the advent of sequencing technologies, metabolomics technologies are now rapidly emerging (El Amrani et al., 2015; Daliri et al., 2017; Parmar et al., 2017). Though in the current analysis environmental approximations are based on computational predictions, we expect that in the very near future a growing number of ecosystems will be subject to an extensive profiling by metabolomics technologies. The framework was designed to allow the future integration of such data in concert with ultra-high coverage metagenomic sequencing. Finally, the inclusion of transcriptomic data, produced together with metabolomics and higher coverage metagenomic information will allow a more comprehensive and more accurate description of community function. Information on transcriptomics/metabolomics paves the way for quantitative predictions of metabolic fluxes (Heinken and Thiele, 2015; Sajitz-Hermstein et al., 2016; Valgepea et al., 2017). To date, quantitative modeling using for example, Constraint-Based Modeling is typically applicable to relatively simplistic communities and consortia (Ye et al., 2014; Koch et al., 2016; Budinich et al., 2017). Recent works attempt to apply quantitative models toward the study of complex microbial communities (Bauer et al., 2017; Magnusdottir et al., 2017). The partiality of data (metagenomics, metatranscriptomics, and metabolomics) from highly diverse ecosystems, together with the computational complexity associated with community-level genome scale metabolic modeling and biases stemming from automated and semi-automated model curation approaches makes topological-based qualitative approaches, as applied here, a powerful and relatively straightforward framework for the analysis of genome-wide ‘omics’ data (Heinken and Thiele, 2015; Taxis et al., 2015; Charitou et al., 2016). Furthermore, it has been suggested that ecological dynamics, as predicted by network topology based frameworks, are of great impact on the metabolic capacity of complex bacterial communities and provide insights on the drivers of species-metabolite dynamics (Noecker et al., 2016). Though predictions derived from the framework might include biases introduced due to the limitations of the current data, many of our simulated observations correspond with the documented role of bacterial groups, supporting the biological relevance of the analyses. Such predictions should be treated as educated ‘leads’ that are useful for the formulation of testable hypotheses. Predictions from the framework used here allow researchers to delineate biological signal from complex data and to rationally design possible manipulation strategies that will induce optimized function. Predictions-based design of agricultural practice can include (i) the identification of microorganisms carrying desired or undesired functions and (ii) the characterization of the effect of the introduction of environmental treatments (that is, adding/depleting specific compounds) (Mazzola and Freilich, 2017). In the absence of appropriate analysis tools and considering the volume of data produced in metagenomics studies, identification of meaningful associations resembles finding a needle in a haystack. Hence, despite limitations, metabolic models can serve as a starting point for generating experimentally testable hypotheses (Magnusdottir et al., 2017).

In summary, this work contributes to the current efforts in the field of Systems Biology for developing new conceptual approaches for the analyses of metagenomic data allowing delineating biological processes and integrating testable predictions. More generally, the framework addresses key ecological challenges regarding the intricate associations between community structure, community function and metabolic inputs and is applicable to a wide array of systems including the human gut, biofilms biotechnological production and bioremediation.

## Author Contributions

SO and SF have substantially contributed to the conception or design of the work, drafting and writing of the manuscript and figures. MO-L and NS substantially contributed to the preparation, collection and analysis of all data. JJ, DM, and YK have contributed to the manuscript and provided mentoring guidance throughout the working process. All authors agree to be accountable for all aspects of the work in ensuring that questions related to the accuracy or integrity of any part of the work are appropriately investigated and resolved.

## Conflict of Interest Statement

The authors declare that the research was conducted in the absence of any commercial or financial relationships that could be construed as a potential conflict of interest.

## Supplementary Material

FIGURE S1 Venn diagrams showing the distribution of differentially abundant enzymes **(A)**, source-metabolites **(B)** and predicted network metabolites **(C)**, in cucumber versus wheat environments.

FIGURE S2 Schematic illustration of the process of source metabolite selection and network reconstruction. The networks are comprised from metabolites denoted by circles that are connected by reactions (edges). A metabolite that is considered as a seed is colored black. **(A)** Identification of source-metabolites in the meta-network, containing all cross-sample reactions. **(B)** Network reconstruction and seed discovery for four networks describing the root and soil environments of each crop. Each network contains all the differentially abundant enzymes in the corresponding environment. Common seeds between a specific environments and the initial meta-network are denoted black with a colored rim. Green shades represent root environments, brown/orange shades represent soil environments. Colored circles are source-metabolites identified only for the network of differentially abundant enzymes and not for the meta-network and are likely to represent biases formed from the gapped nature of a network which relies solely on differentially abundant enzymes. **(C)** The common seeds between the meta-network and each environment represents the predicted specific environment and was further used for the meta-network expansion.

FIGURE S3 Simpson and Shannon index values distribution across environments. **(A)** Spearman correlation matrix (*p* < 0.05) between the Simpson and Shannon indices, showing similarity between replicas and negative correlations between diversity and dominance values. For each index, soil and root samples are co-clustered together. **(B)** Scatter plots of data per sample. The colored areas represent the cut-off chosen for high dominance and low diversity data taken into further analysis. Green hues represent the root data and brown the soil data.

FIGURE S4 A schematic illustration of the possibility of complementary metabolites per a combination of key taxonomic groups. Nodes represent metabolites and edges represent reactions. **(A)** A network constructed from a core enzyme set, existing in all key taxonomic groups. **(B)** Network constructions of the core enzyme set augmented with enzymes unique to taxonomic groups A or B, added metabolites are colored green or blue respectively. **(C)** A network construction of the core enzyme set with the addition of unique enzymes from both taxonomic groups A and B at once. Green or blue metabolites represent metabolites specific to either taxonomic group (A or B). Red metabolites are complementary (new) metabolites, present only for the combination of taxonomic groups A and B.

FIGURE S5 General pathway category distribution of enzymes dominated by specific taxonomic groups.

FIGURE S6 Distribution of enzymes dominated by taxonomic groups across environments. The top five taxonomic groups in each environment were defined as the key taxonomic groups (per environment).

TABLE S1 Differential abundance analysis of cucumber root and soil samples.

TABLE S2 Differential abundance analysis of wheat root and soil samples.

TABLE S3 Metabolites predicted as describing the environments of cucumber.

TABLE S4 Metabolites predicted as describing the environments of wheat.

TABLE S5 Count of network metabolites and reactions in the four environment-specific networks.

TABLE S6 Simpson and Shannon indices calculations per enzyme per environment.

TABLE S7 Environment level associations between enzyme and taxonomy.

TABLE S8 Key taxonomic groups combinations taken for synergistic complementation calculations. Six total key taxonomic groups were taken into account for investigation. Core metabolites common to every group were included in every combination.

TABLE S9 Complementary (synergistic) metabolites mapping to pathways.

TABLE S10 Pathway distribution of differential abundant enzymes (**Figure 1A**, top). Pathway enrichment analysis by the Hypergeometric distribution test and goodness of fit chi square test (*p-*value < 0.05 highlighted in green and yellow respectively).

TABLE S11 Pathway distribution of source metabolites (**Figure 1A**, middle). Pathway enrichment analysis by the Hypergeometric distribution test and goodness of fit chi square test (*p*-value < 0.05 highlighted in green and yellow respectively).

TABLE S12 Pathway distribution of network metabolites (**Figure 1A**, bottom). Pathway enrichment analysis by the Hypergeometric distribution test and goodness of fit chi square test (*p*-value < 0.05 highlighted in green and yellow respectively).

TABLE S13 Taxonomic mapping of dominated enzymes. Distribution of dominated enzymes between taxonomic groups in different environments.

TABLE S14 Impact of dynamic removal of key taxonomic groups, one by one and all at once, from the meta-network. The impact is calculated as the metabolite number difference between the original and removed network. Enzymes specific to a key taxonomic group were removed from the general metagenomic enzyme set dynamically. Next, the reduced enzyme set was expanded into four networks, one per environment.

TABLE S15 Effect of the removal of key taxonomic groups in the wheat root environment. The effect score is calculated as the pathway coverage difference between the removed network and the original.

TABLE S16 Effect of the removal of key taxonomic groups in the cucumber root environment. The effect score is calculated as the pathway coverage difference between the removed network and the original.

TABLE S17 Effect of the removal of key taxonomic groups in the wheat soil environment. The effect score is calculated as the pathway coverage difference between the removed network and the original.

TABLE S18 Effect of the removal of key taxonomic groups in the cucumber soil environment. The effect score is calculated as the pathway coverage difference between the removed network and the original.

TABLE S19 Number of complementary (synergistic) metabolites per combination per environment. Shade intensity increases with metabolite number.
