# Supplementary figures and images for: Analysis of Microbial Functions in the Rhizosphere Using a Metabolic-Network Based Framework for Metagenomics Interpretation
Source: Front Microbiol. 2017 Aug 23;8:1606. doi: 10.3389/fmicb.2017.01606 (PMC5572346; doi:10.3389/fmicb.2017.01606)

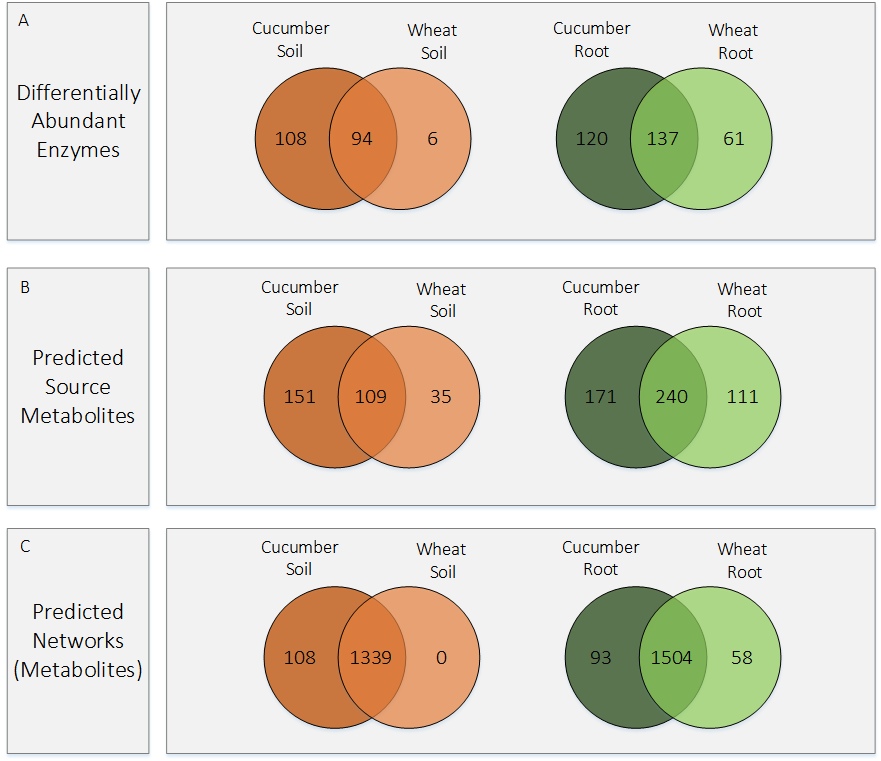

Supplement: FIGURE S1 — Venn diagrams showing the distribution of differentially abundant enzymes (A), source-metabolites (B) and predicted network metabolites (C), in cucumber versus wheat environments. [file Image_1.TIF]

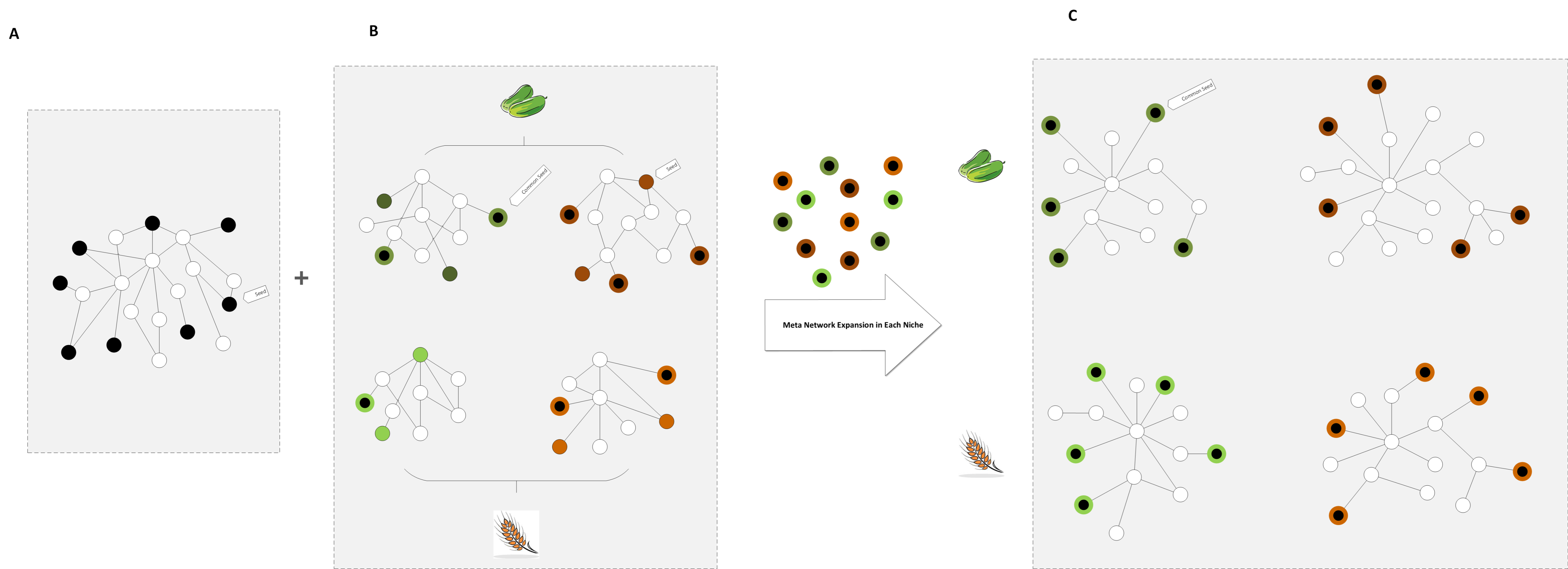

Supplement: FIGURE S2 — Schematic illustration of the process of source metabolite selection and network reconstruction. The networks are comprised from metabolites denoted by circles that are connected by reactions (edges). A metabolite that is considered as a seed is colored black. (A) Identification of source-metabolites in the meta-network, containing all cross-sample reactions. (B) Network reconstruction and seed discovery for four networks describing the root and soil environments of each crop. Each network contains all the differentially abundant enzymes in the corresponding environment. Common seeds between a specific environments and the initial meta-network are denoted black with a colored rim. Green shades represent root environments, brown/orange shades represent soil environments. Colored circles are source-metabolites identified only for the network of differentially abundant enzymes and not for the meta-network and are likely to represent biases formed from the gapped nature of a network which relies solely on differentially abundant enzymes. (C) The common seeds between the meta-network and each environment represents the predicted specific environment and was further used for the meta-network expansion. [file Image_2.TIF]

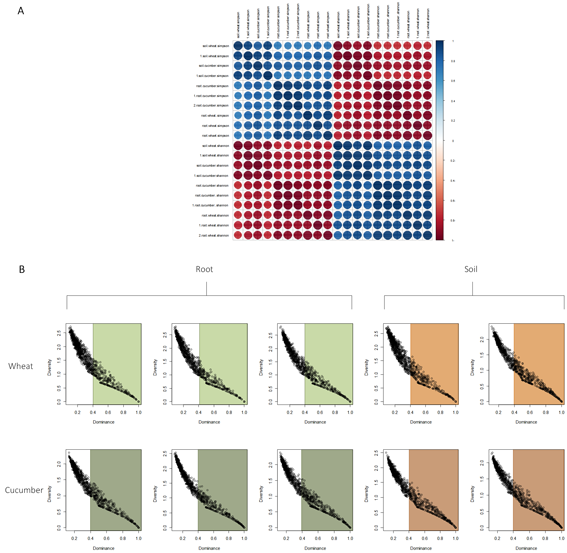

Supplement: FIGURE S3 — Simpson and Shannon index values distribution across environments. (A) Spearman correlation matrix (p < 0.05) between the Simpson and Shannon indices, showing similarity between replicas and negative correlations between diversity and dominance values. For each index, soil and root samples are co-clustered together. (B) Scatter plots of data per sample. The colored areas represent the cut-off chosen for high dominance and low diversity data taken into further analysis. Green hues represent the root data and brown the soil data. [file Image_3.TIF]

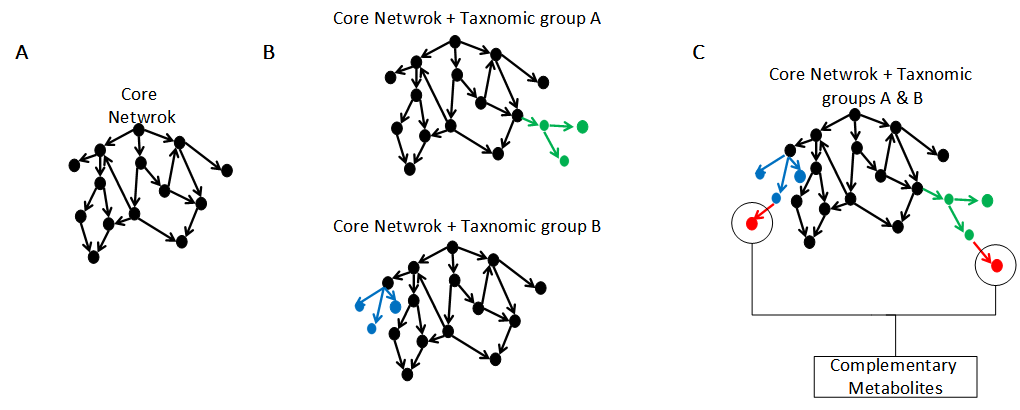

Supplement: FIGURE S4 — A schematic illustration of the possibility of complementary metabolites per a combination of key taxonomic groups. Nodes represent metabolites and edges represent reactions. (A) A network constructed from a core enzyme set, existing in all key taxonomic groups. (B) Network constructions of the core enzyme set augmented with enzymes unique to taxonomic groups A or B, added metabolites are colored green or blue respectively. (C) A network construction of the core enzyme set with the addition of unique enzymes from both taxonomic groups A and B at once. Green or blue metabolites represent metabolites specific to either taxonomic group (A or B). Red metabolites are complementary (new) metabolites, present only for the combination of taxonomic groups A and B. [file Image_4.TIF]

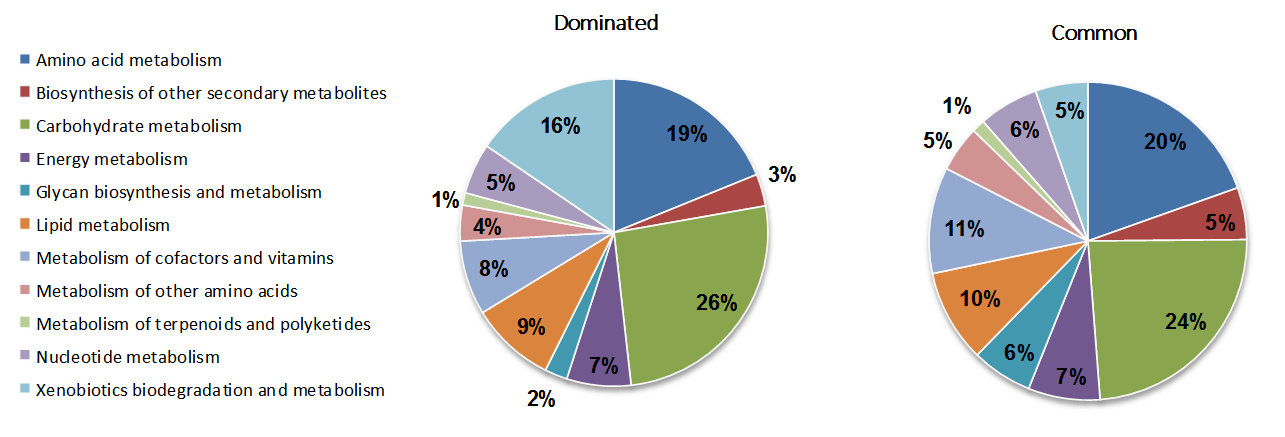

Supplement: FIGURE S5 — General pathway category distribution of enzymes dominated by specific taxonomic groups. [file Image_5.TIF]

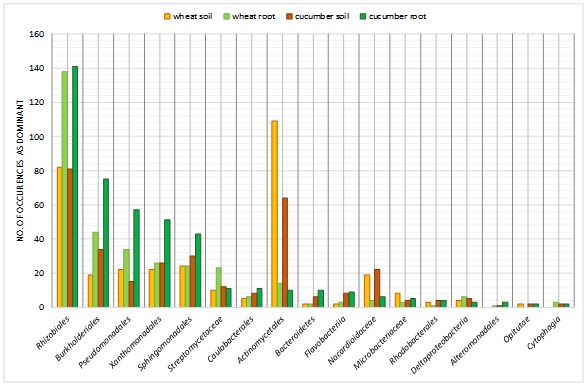

Supplement: FIGURE S6 — Distribution of enzymes dominated by taxonomic groups across environments. The top five taxonomic groups in each environment were defined as the key taxonomic groups (per environment). [file Image_6.TIF]
